# Supplementary material for: MetaRibo-Seq measures translation in microbiomes
Source: Nat Commun. 2020 Jun 29;11:3268. doi: 10.1038/s41467-020-17081-z (PMC7324362; doi:10.1038/s41467-020-17081-z)
Supplement: Supplementary file 10 — Supplementary Data 7 [file 41467_2020_17081_MOESM10_ESM.zip › File2/Confidence_VeryHigh_Taxonomy/164215_out.krona.html]

Javascript must be enabled to view this page.

members
magnitude
magnitudeUnassigned
count
unassigned
taxon
rank

164215\_out

6

2

SRS076929\_contig\_number\_contig-100\_17846.109081SRS1041145\_contig\_number\_30833

2
4
superkingdom

4
phylum
1239

186801
class
4


SRS023914\_contig\_number\_26531SRS051031\_contig\_number\_26394SRS098571\_contig\_number\_67658
order
3
4
186802

541000
1
family

946234
genus
1

1
species

SRS058070\_contig\_number\_contig-100\_2563.86942
1193534
